# Cytotoxic effects of dental prosthesis grinding dust on RAW264.7 cells

**DOI:** 10.1038/s41598-020-71485-x

**Published:** 2020-09-01

**Authors:** Wei Wang, Tianshu Li, Xue Luo, Ke Zhang, Nanjue Cao, Keda Liu, Xiaoming Li, Yuhe Zhu

**Affiliations:** 1 School and Hospital of Stomatology, China Medical University, Liaoning Provincial Key Laboratory of Oral Diseases, Shenyang, 110002 China; 2 Key Laboratory for Biomechanics and Mechanobiology of Ministry of Education, School of Biological Science and Medical Engineering, Beihang University, Beijing, 100083 China; 3 Beijing Advanced Innovation Center for Biomedical Engineering, Beihang University, Beijing, 100083 China; 4 The Fourth Affiliated Hospital, Zhejiang University School of Medicine, Yiwu, 322000 China

**Keywords:** Occupational health, Inflammasome, Inflammasome, Inflammasome, Occupational health

## Abstract

Respiratory diseases, including pulmonary fibrosis, silicosis, and allergic pneumonia, can be caused by long-term exposure to dental prosthesis grinding dust. The extent of the toxicity and pathogenicity of exposure to PMMA dust, Vitallium dust, and dentin porcelain dust differs. The dust from grinding dental prosthesis made of these three materials was characterized in terms of morphology, particle size, and elemental composition. The adverse effects of different concentrations of grinding dust (50, 150, 300, 450, and 600 μg ml^−l^) on RAW264.7 macrophages were evaluated, including changes in cell morphology and the production of lactate dehydrogenase (LDH) and reactive oxygen species (ROS). The dust particles released by grinding dental prosthesis made of these materials had different morphologies, particle sizes, and elemental compositions. They also induced varying degrees of cytotoxicity in RAW264.7 macrophages. A possible cytotoxicity mechanism is the induction of lipid peroxidation and plasma membrane damage as the dust particles penetrate cells. Therefore, clinicians who regularly work with these materials should wear the appropriate personal protection equipment to minimize exposure and reduce the health risks caused by these particulates.

## Introduction

Dental prostheses are made and tested, a variety of dust particles are emitted from the grinding performed to correct defects^1–4^. Commonly used prosthetic materials include PMMA, Vitallium, and porcelain, and the dust created from grinding them may pose various health hazards to oral care workers, such as pulmonary fibrosis, silicosis, allergic pneumonia, lung granuloma, asthma, and lung cancer^5–8^.

Epidemiological investigations indicated that the effects of dental prosthesis grinding dust on the respiratory system of oral care workers may be correlated with dust exposure time and the type of dust. In addition, many studies have reported that the type of abrasive dust is closely related to specific respiratory diseases of oral care workers^9–13^. Zhang et al. reported that animals were subjected to mixed dust types generated from grinding dental prostheses (PMMA, Vitallium and porcelain dust, etc.), and then, the histopathology of their lung tissue was examined. It was proposed that grinding dust induced fibrosis in rat lung tissue^14^. Upadhyay et al. found that rats exposed to three different quality levels of fine dust (including 250, 500 and 1,000 μg ml^−l^) presented with increases in the total number of inflammatory cells and levels of interleukin-6^15^.

RAW264.7 cells are mouse mononuclear macrophage leukaemia cells that play key roles in inflammation, immunity and phagocytosis. They are readily available and their use raises fewer ethical issues than using human macrophages. The macrophage pneumoconiosis model is a currently established model^16^. Various particles produce similar results. When calcium carbonate, silica dust, etc., are applied to cells, reactive oxygen species (ROS) and lactate dehydrogenase (LDH) are commonly measured as indicators of cytotoxicity. Yang et al. reported that macrophages produce ROS after engulfing dust particles^17^. The large amount of ROS can produce cell lipid peroxidation, which damages normal cells and tissues^18^. LDH is a sensitive indicator of cell membrane changes. Measures of LDH activity can reflect changes in membrane permeability and damage of RAW264.7 cells^19^. Currently, there are few reports on the effects of prosthesis grinding powder in vivo. Some researchers have collected mixed dust obtained from oral prosthesis grinding and expose rats to it, and they observed pathological sections of rat lung tissue, suggesting that the exposure to the dust had a fibrosis-inducing effect on rat lung tissue^20^. Wen et al. reported that the toxicity of hydrogen peroxide (H_2_O_2_) was evaluated by detecting the levels of ROS and LDH in RAW264.7 cells^21^.

In this study, the morphologies, particle size distributions, and compositions of the three types of dental prosthesis grinding dust were measured. The effect of these dust particles at different concentrations on RAW264.7 cells was evaluated. This information may provide a basis for determining the best personal protection for use by dental clinicians and guide follow-up research.

## Results

### Particle characterization

**Figure 1:**
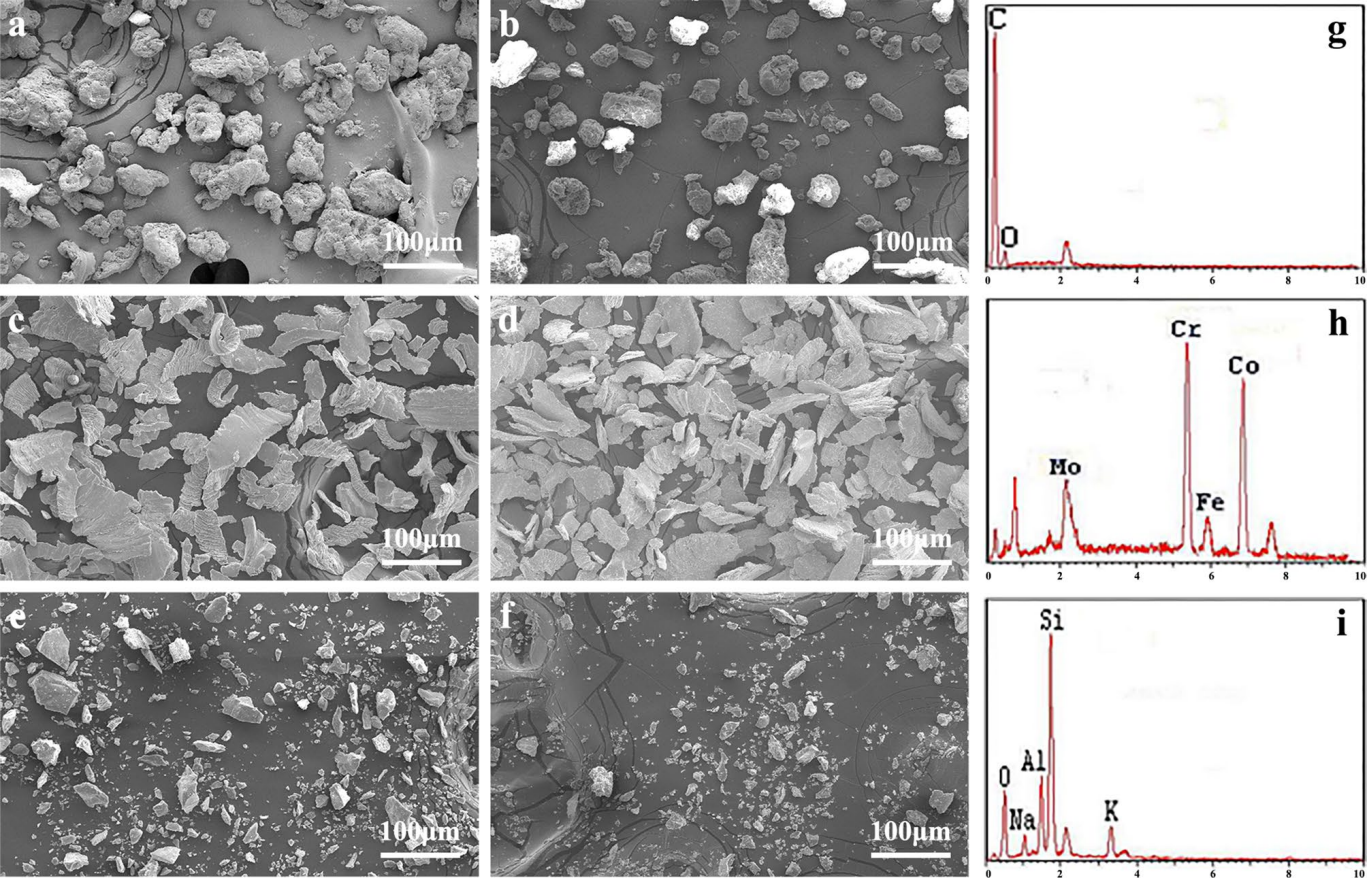
Scanning electron microscopy images of dental prosthesis grinding dust: PMMA (**a**), finely ground PMMA (**b**), Vitallium (**c**), finely ground Vitallium (**d**), porcelain (**e**), finely ground porcelain (**f**), and their composition: PMMA (**g**). Vitallium (**h**), porcelain (**i**).

The surface morphologies of the grinding dust were examined by SEM before and after fine milling. The results showed that the three types of grinding dust had entirely different morphologies. As shown in Fig. 1, the morphology of the PMMA group particles (a, b) was irregular and very different in size. The Vitallium group (c, d) consisted of particles arranged in a sheet with irregular edge morphology. The porcelain group (e, f) consisted of cube-shaped particles with sharp edges. After fine milling, only the particle sizes were reduced, but their shapes did not significantly change.

Table 1 shows detailed information on the particle sizes of the three grinding dust types before and after fine milling. The three types of grinding dust particles showed had wide range of size distributions that significantly differed from each other. Fine grinding reduced the particle sizes to a different extent for each dust type. Furthermore, 42.3% of the finely ground porcelain dust particles were smaller than 10 μm, which is a higher than the proportions of particles in the same size range for the other types of grinding dust.

**Table 1:** Particle size distribution of the three dental prosthetic grinding dust particles before and after fine milling.

	A-1	A-2	B-1	B-2	C-1	C-2
< 5 μm (%)	4.19	8.23	0.08	1.03	12.79	21.05
5–10 μm (%)	1.57	4.3	0.62	2.37	12.74	21.25
> 10 μm (%)	94.24	87.47	99.3	96.6	74.47	47.7

*A-1* PMMA, *A-2* finely ground PMMA, *B-1* Vitallium, *B-2* finely ground Vitallium, *C-1* porcelain, *C-2* finely ground porcelain.

EDX was used to determine the compositions of the three types of dental prosthesis grinding dust particles. As shown in Fig. 1, PMMA dust (g) contained mainly C and O, Vitallium dust (h) was composed mostly of Cr, Co, and Mo, and porcelain dust (i) consisted principally of Si, Al, and O.

### Cell viability

**Figure 2:**
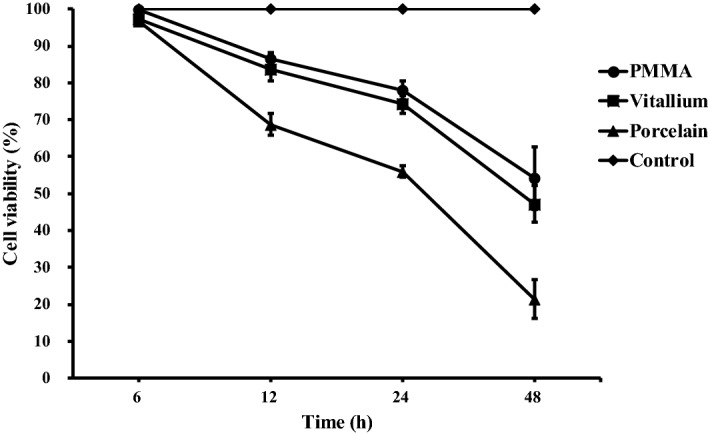
Cell viability rate of 300 μg mL^−l^ of different types of dental prosthesis grinding dust particles on RAW264.7 cells after 24 h of exposure.

The viability of the RAW264.7 cells exposed to the three kinds of dust was lower than that of the blank control group cells (Fig. 2). The viability of the RAW264.7 cells was inversely proportional to the dust exposure time. Compared with the control group at different time points, the cell survival rate of the exposed cells gradually decreased. At 6 h, the number of viable exposed cells was reduced, but the change was not significant (*P* > 0.05). Twelve, twenty-four and forty-eight hours after dust treatment, cell viability was significantly reduced. Compared with the control group, the difference was statistically significant (*P* < 0.01). There was no significant difference between the PMMA group and the Vitallium group, and the porcelain group was significantly different from both other groups (*P* < 0.05).

### In vitro toxicology assays

**Figure 3:**
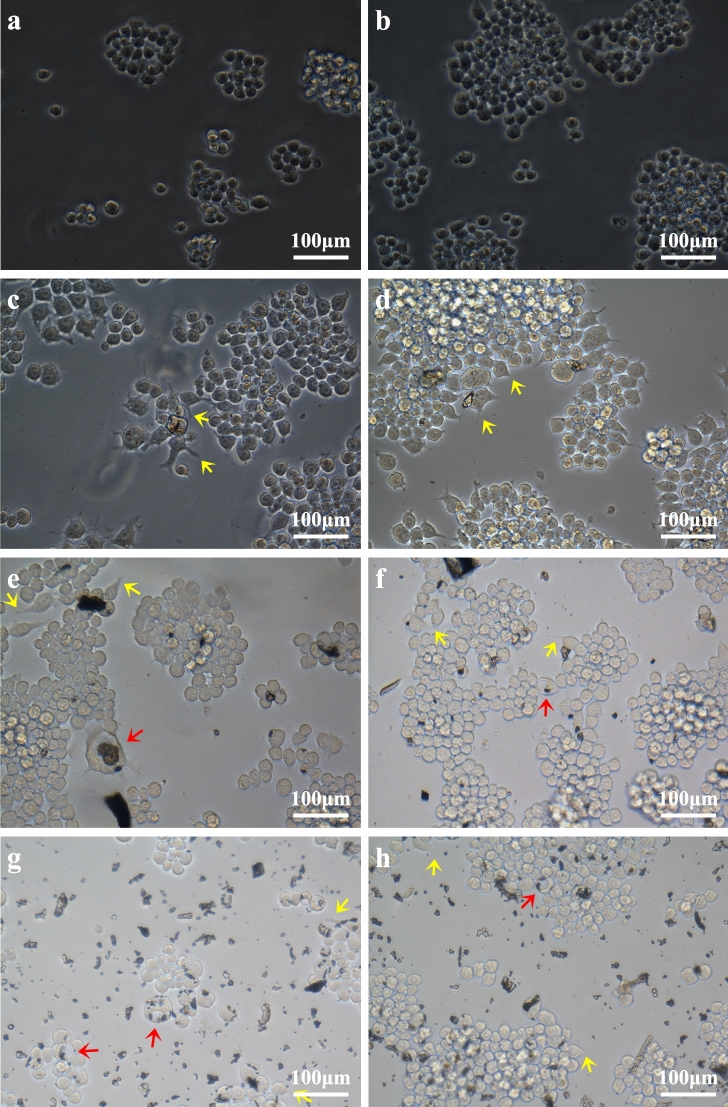
Effects of 300 μg mL^−l^ of the different types of dental prosthesis grinding dust particles on RAW264.7 after 24 h of exposure: PMMA (**a**), Vitallium (**c**), porcelain (**e**), and the negative control (**g**); after 48 h, PMMA (**b**), Vitallium (**d**), porcelain (**f**), and the negative control (**h**). The yellow arrow indicates the pseudopod extension, and the red arrow indicates the balloon change.

**Figure 4:**
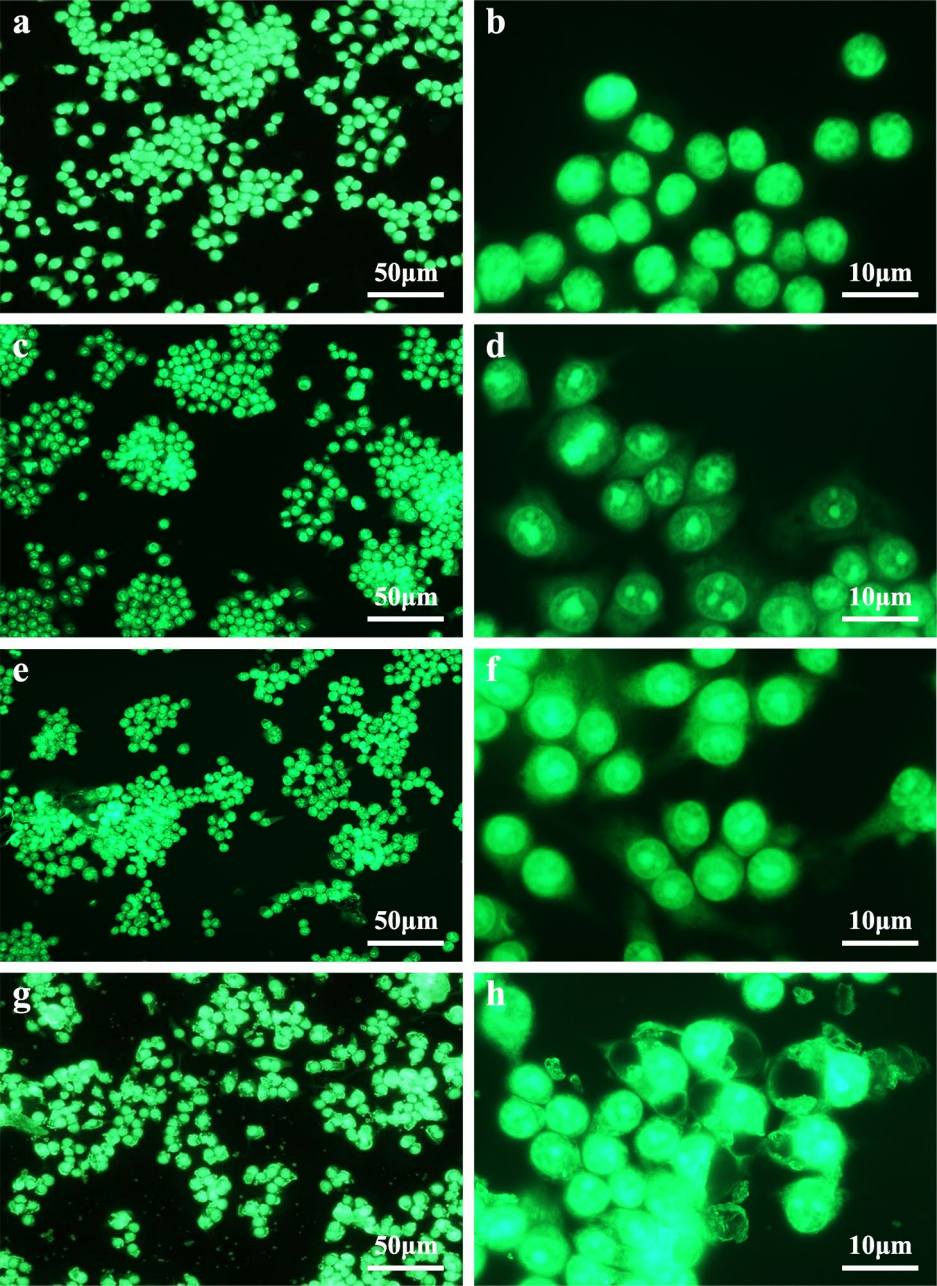
Fluorescent staining of RAW264.7 cells exposed to 300 μg mL^−l^ dust from grinding different types of dental prosthesis materials: negative control (**a**,**b**), PMMA (**c**,**d**), Vitallium (**e**,**f**), and porcelain (**g**,**h**).

Light and fluorescence microscopy (Fig. 3) showed that after the three types of prosthesis materials were ground, the grinding dust remained in contact with the RAW264.7 macrophages. A phase-displaced microscope was used to observe the morphological changes of the RAW264.7 cells after 24 h and 48 h of exposure at a concentration of 300 μg ml^−1^. The negative control cells showed a regular circular shape, but the surface of the cells exposed to prosthesis grinding dust was changed. After 24 h and 48 h of cultivation, the dust adsorbed onto the cell surface or entered the cells. The cells showed irregular shapes, the number of pseudopods was increased, the pseudopods were elongated to different lengths, and some cells even appeared balloon-like. Under low magnification (200 ×), as shown in Fig. 4a,c,e,g, no abrasive grinding dust was found to induce apoptosis of the RAW264.7 cells. Furthermore, under high magnification (400 ×), as shown in Fig. 4b,d,f,h, the pseudopods of the cells were elongated, and the nuclei were displaced. In particular, the changed in nuclear location was more pronounced in the cells exposed to the porcelain dust.

**Figure 5:**
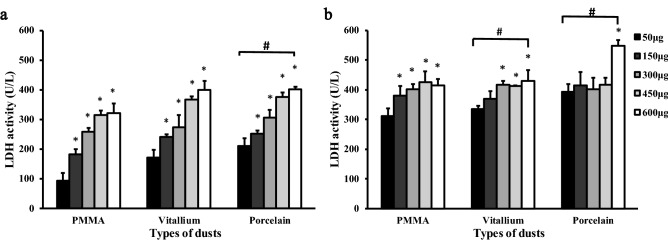
LDH activity in RAW264.7 macrophages exposed to different types of dental prosthesis grinding dust for 24 h (**a**) and 48 h (**b**).

**Figure 6:**
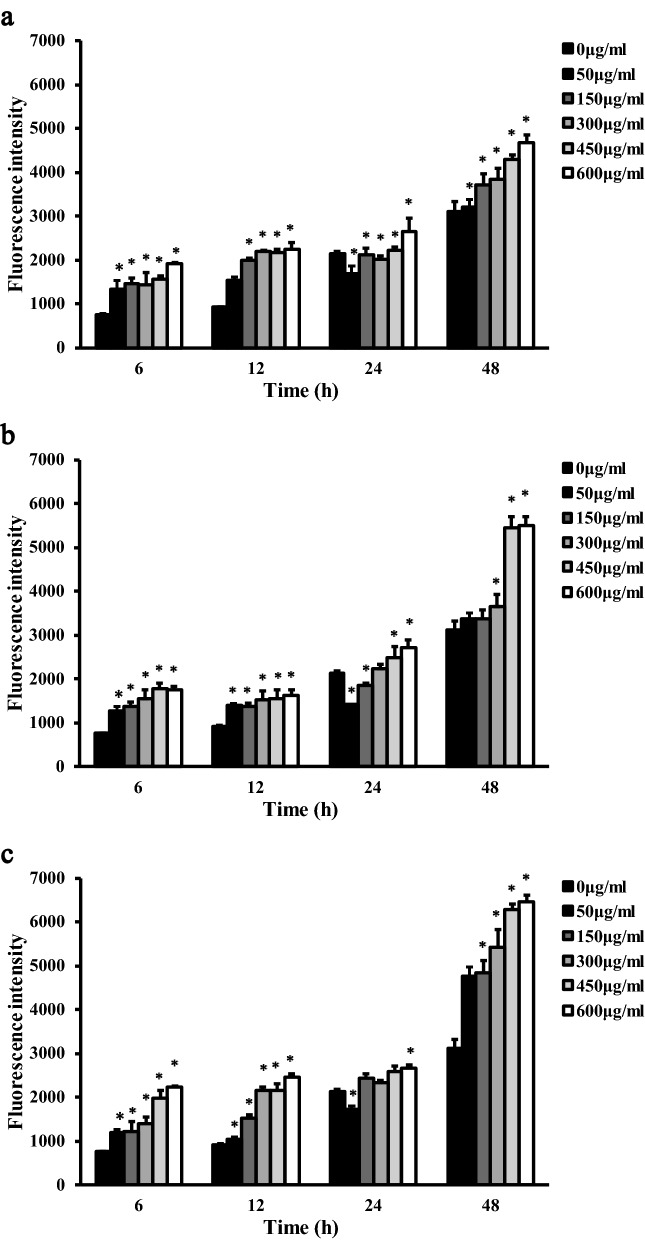
Comparison of ROS levels in RAW264.7 macrophages exposed to five different concentrations of PMMA dust (**a**), Vitallium dust (**b**), and porcelain dust (**c**) for 6 h, 12 h, 24 h, and 48 h.

The results of LDH and ROS fluorescence intensity detection are shown in Figs. 5 and 6 The data were subjected to multiple linear regression and showed that the order of influence of the factors on LDH and ROS was the time of exposure to the dust, the dust concentration, and the dust type. The amounts of LDH and ROS released were increased with time and concentration, and the differences were statistically significant (*P* < 0.05). LDH activity was observed, in descending order, for the porcelain dust group, Vitallium group and PMMA group. At 24 h and 48 h, the difference between the porcelain dust group and the PMMA group was statistically significant (*P* < 0.05), and the difference between the Vitallium group and the PMMA group at 48 h was statistically significant (*P* < 0.05). At 24 h, the ROS fluorescence intensity of the three dust samples was slightly lower than that of the negative control group. The difference between the three dust samples and the negative control group was statistically significant (*P* < 0.05). In addition, the multiple comparison analysis based on LSD showed that the effect of the porcelain dust group was higher than the PMMA group and the Vitallium group, and the difference was statistically significant (*P* < 0.05). Moreover, there was no significant difference between the Vitallium group and the PMMA group.

## Discussion

Grinding dust toxicities were closely correlated with their physicochemical properties, including particle diameter, shape, surface area, and constituent elements^22,23^. The size of the particle has various effects on the organisms exposed to them. Dust particles > 10 μm enter through the nasal cavity and are deposited mainly in the back of the pharynx^24^. Particles 5–10 μm in diameter are deposited in the large and middle airways. Particles < 5 μm in diameter can damage alveolar macrophages and cause silicosis, pulmonary fibrosis, and other respiratory diseases^25^. Researchers found that RAW macrophage cells effectively internalized both 0.1- and 0.5-μm-diameter dust particles. After 6 h of exposure, numerous cells had extended pseudopods, indicating active removal of particles^26^. Through TEM analysis, researchers found that dust particles can enter human bronchial epithelial cells. Particles can usually be observed in the form of clumps in intracellular vesicles in the cytoplasm. Even in cases for which the particles are not clearly observed in the nucleus, in the presence of large vesicles, the shape of the nucleus appears abnormal and compressed^27^. Previous animal experiments showed that the relatively smaller particle sizes of porcelain and Vitallium dust particles may be more toxic than larger particles^15^. This finding was consistent with the results of the present study. Among the three types of denture grinding dust, the percentage of ceramic abrasive grains with a diameter of < 5 μm was the highest (21.05%); therefore, porcelain powder may cause more lung damage than is caused by other dust types. In addition, the percentage of PMMA abrasive dust particles < 5 μm was higher than that of the Vitallium abrasive dust. However, the experimental results showed that Vitallium abrasive dust caused slightly greater damage to the RAW264.7 cells, but the difference was not statistically significant and may be related to the dust particle structures, element compositions or way of entering the body.

With increasing dust exposure time, the cell survival rate decreased. After 12 h, the effect of the porcelain powder on cell proliferation activity was significantly different that it was on the proliferation of the other two groups. Microscopic observation revealed that the dust particles accumulated on the RAW264.7 macrophage surfaces, which caused pseudopod elongation, irregular cell morphology, and ballooning. Particle binding to cell surface receptors such as scavenger and Toll-like receptors may induce polymerization of the actin cytoskeleton and the formation of pseudopods to envelop the dust particles^28^. Particles engulfed by macrophages are transported in vesicles. These phagosomes mature and bind to lysosomes which digest the particles with enzymes such as hydrolase and oxidase^29^. This process damages cells, releases dust particles, initiates re-phagocytosis, and stimulates the proliferation and aggregation of macrophages. All these events can trigger an inflammatory response^30^.

The effects of dust on macrophages via the classical (type 1 helper T cell factor involvement) and selective activation (type 2 helper T cell factor involvement) pathways^31^. The classical activation pathway generates numerous inflammatory cytokines and free radicals^32,33^. For example, Sato et al. reported that macrophages exposed to silica dust activated classical pathways and therefore generated superoxide anion (O_2_^−^) and hydroxyl (OH^−^), which caused oxidative damage to the cells^34^. In addition, free radicals can lead to lipid peroxidation of the cell membrane^35^. This study focused on the classical pathway. All three types of fine dental prosthesis grinding dust led to ROS increases in the macrophages in a time-dependent manner. The porcelain dust induced higher levels of ROS than either the PMMA or Vitallium dust. Therefore, porcelain caused a severe lipid peroxidation reaction, and the LDH level increase induced by porcelain exposure was consistent with the ROS level increase induced by porcelain. Twenty-four hours after low-concentration dust was applied to RAW264.7 cells, the ROS content level decreased slightly compared with that of the negative control group, which may have been the result of differences in the degree of immune repair regulation caused by the dust-induced damage to the cells^36^. However, with the further increases in concentration, the ROS content levels showed an increasing trend, which may indicate that the cells could not repair the damage caused by exposure to high concentrations of dust^37^. Arnoldussen et al. showed that the release of ROS from astrocytes increased with increasing concentration, but these cells did not produce ROS when exposed to a low concentration level (0.0009 μg/cm^2^) for 72 h^38^. The ROS levels increased significantly, similar to the results of this experiment. The reason for the ROS increase might be related to short-term and low-dose dust exposure initiating defence measures in the body. However, the grinding dust concentrations used in this experiment do not necessarily represent the actual concentrations of grinding dust that would actually enter the lungs during occupational exposure^39^.

## Conclusions

Three types of dental prosthesis grinding dust particles, PMMA dust, Vitallium dust, and dentin porcelain dust, were all toxic to RAW264.7 cells. The toxicity of the porcelain dust was greater than that of the Vitallium dust or PMMA dust, and the cytotoxicity increased with increasing dust exposure time and concentration. The possible mechanism of toxicity may relate to the manner in which the dust entered the cell and its production of lipid peroxidation, which damaged the cell membrane and other structures. Therefore, the occupational protection of the clinician should be increased to reduce the damage of the dust to the doctor.

## Materials and methods

### Dental prosthesis grinding dust preparation

PMMA, Vitallium, and porcelain test modules were prepared as per clinical procedures. The dust was sealed in containers with the emery burrs. Ten grams of each dust type was weighed. The three types of dust particles were allocated to groups A, B, and C, which in turn were divided into two subgroups, namely, A1, A2, B1, B2, C1, and C2. A1, B1, and C1 were untreated samples. A2, B2, and C2 were finely ground by an agate mortar for 2 h, and their morphologies, sizes, chemical compositions, and cytotoxicity were examined.

### Particle characterization

Scanning electron microscopy (SEM; SSX-550y, Shimadzu, Japan) was used to observe the morphology of the dental prosthesis grinding dust. The distribution of particle size was measured by a particulate size description analyser (PSDA; Mastersizer Macro, Malvern, United Kingdom). Energy dispersive spectroscopy (EDX; Shimadzu, Japan) was used to analyse the elemental compositions of these dust samples.

### Cell viability test

RAW264.7 macrophages were selected in logarithmic phase, adjusted to 1 × 10^6^ cells/ml and inoculated in 96-well plates, with 200 μl of the cell suspension added to each well, and cultured in a 37 °C in an incubator with 5% CO_2_. After 24 h, the culture medium was discarded, and a 300 µg/ml suspension of each of the three kinds of dust particles was added to a volume of 200 µl in each well for the exposure test. After exposure for 6, 12, 24, and 48 h, 10 μl of MTT reagent (Sigma-Aldrich) was added to each well and incubated for 4 h. Then, the supernatants were removed, 200 μl of DMSO was added, and the 96-well plate was shaken on a horizontal shaker for 10 min. The absorbance measurement of the wells without dust suspension were used as the reference points for the absorbance measurement of the exposed cells, as determined by a microplate reader (Bio Tek, Winooski, VT) at 490 nm. Cell viability was tested by the MTT method.

### Toxicology

The cytotoxicity induced in RAW264.7 macrophages by the exposure to different concentrations (50, 150, 300, 450, and 600 μg mL^−l^) of dust suspensions in DMEM containing 10% inactivated FBS was observed by light and fluorescence microscopy. It was also evaluated with LDH and ROS assays. Coverslips were autoclaved, placed in a six-well plate (1 × 10^5^ cells/well), and exposed to 2 mL of 300 μg ml^−l^ grinding dust suspension in 5% CO_2_ at 37 °C for 24 h and 48 h. The coverslips were then removed and placed onto clean glass slides. These cell samples were observed under an inverted phase-contrast microscope. The cells were stained by fluorescent dye by adding 1 ml of 95 v/v % alcohol to each well and, 30 min later, followed by the addition of 0.1 mg ml^−l^ acridine orange and incubation for 15 min in the dark at room temperature. Finally, the cells were gently rinsed with PBS and observed under a fluorescence microscope.

RAW264.7 macrophages were seeded in a 96-well plate (1 × 10^4^ cells/well) and exposed to 200 μl of the grinding dust suspension at concentrations of 50, 150, 300, 450, and 600 μg ml^−l^. Three wells were designated for each type of dust. Complete culture medium (DMEM supplemented with 10% inactivated serum) was used as a negative control. The cells were incubated in 5% CO_2_ at 37 °C for 6, 12, 24, and 48 h. However, the incubation times were only 24 h and 48 h for the LDH analysis. Two hundred microlitres of 2′,7′-dichlorofluorescein-diacetate (DCFH-DA) fluorescent probe (10 μM) was added to each well, and the cells were incubated in 5% CO_2_ at 37 °C for 1 h. Residual DCFH-DA was then removed by rinsing the cells three times with PBS. Then, 100 μL of PBS was added to each well, and the plates were placed on a microplate reader (Infinite M200, Tecan, Austria), and the OD values of each were measured at 488 nm and 525 nm. The average fluorescence intensities were calculated for the various concentrations of each grinding dust. At 24 h and 48 h, culture supernatants were sampled from each well and analysed for LDH level using a lactate dehydrogenase kit.

### Data analysis

Statistical analysis was performed with SPSS 17.0 (IBM Corp., Armonk, NY, USA). Multivariate linear regression, one-way ANOVA, and LSD test were used to compare differences between groups. *P* values less than 0.05 were stated as statistically significant.
